# A new item response theory model to adjust data allowing examinee choice

**DOI:** 10.1371/journal.pone.0191600

**Published:** 2018-02-01

**Authors:** Carolina Silva Pena, Marcelo Azevedo Costa, Rivert Paulo Braga Oliveira

**Affiliations:** 1 Department of Production Engineering, Federal University of Minas Gerais, Belo Horizonte, Minas Gerais, Brazil; 2 Pró-Reitoria de Graduação, Federal University of Minas Gerais, Belo Horizonte, Minas Gerais, Brazil; 3 Department of Statistics, Federal University of Ouro Preto, Ouro Preto, Minas Gerais, Brazil; Universidad Rey Juan Carlos, SPAIN

## Abstract

In a typical questionnaire testing situation, examinees are not allowed to choose which items they answer because of a technical issue in obtaining satisfactory statistical estimates of examinee ability and item difficulty. This paper introduces a new item response theory (IRT) model that incorporates information from a novel representation of questionnaire data using network analysis. Three scenarios in which examinees select a subset of items were simulated. In the first scenario, the assumptions required to apply the standard Rasch model are met, thus establishing a reference for parameter accuracy. The second and third scenarios include five increasing levels of violating those assumptions. The results show substantial improvements over the standard model in item parameter recovery. Furthermore, the accuracy was closer to the reference in almost every evaluated scenario. To the best of our knowledge, this is the first proposal to obtain satisfactory IRT statistical estimates in the last two scenarios.

## Introduction

Item response theory (IRT) comprises a set of statistical models for measuring examinee abilities through their answers to a set of items (questionnaire). One of the most important advantages of IRT is allowing comparison between examinees who answered different tests. This property, known as invariance, is obtained by introducing separate parameters for the examinee abilities and item difficulties [1]. The IRT models have optimal properties when all items within a test are mandatory for the examinees to answer. In contrast, if the examinees are allowed to choose a subset of items, instead of answering them all, the model estimates may become seriously biased. This problem has been raised by many researchers [2–8] but still lacks a satisfactory solution.

This problem is an important issue because several studies have provided evidence that choice has a positive impact in terms of educational development [9–12]. That is, these studies indicated that allowing students to choose which questions to answer increases motivation and engagement in the learning process. In a testing situation, allowing choice seems to reduce the concern of examinees regarding the impact of an unfavourable topic [13]. In addition, it has been claimed as a necessary step for improving educational assessment [14, 15].

Furthermore, allowing choice could be used to ameliorate important challenges faced in the application of IRT models. For example, to achieve invariance, the items used in different tests must be calibrated in the same scale. This calibration is usually done by creating a question bank from which the selected items are extracted. Items in the bank were previously calibrated by being exposed to examinees who have similarities to those from whom the tests are intended. Therefore, these items were pre-tested. The pre-test process is typically extremely expensive and time consuming. For example, in 2010, it was reported that the Brazilian government spent approximately US$3.5 million (considering an average exchange rate of R$1.75 per US$1.00 in August 2010) to calibrate items for an important national exam (ENEM). Nevertheless, serious problems were reported during the pre-test, such as the employees of a private high school supposedly making illegal copies of many items and their subsequent release. In addition, the number of items currently available in the national bank was approximately 6 thousand, whereas the ideal number would be between 15 and 20 thousand. All these events were harshly criticized by the mainstream media [16–19]. Recent works have proposed optimal designs for item calibration to reduce costs and time [20, 21]. Still, there is a limit on the number of items that an examinee can properly answer within a period of time. If the examinees are allowed to choose *v* items within a total of *V* items (*v* < *V*) and if satisfactory statistical estimates are provided for all *V* items, the costs of calibration per item are reduced.

This paper presents a new IRT model to adjust data generated using an examinee choice allowed scenario. The proposed model incorporates network analysis information using Bayesian modelling in the IRT model. This paper evaluates the one-parameter logistic model, known as the Rasch model [22], which is the simplest and most widely used IRT model [1]. Future studies will aim to extend the proposed approach to more complex IRT models, such as the two- and three-parameter logistic models.

The results show substantial improvements in the accuracy of the estimated parameters compared to the standard Rasch model, mainly in situations in which examinee choice is different from a random selection of items. To the best of our knowledge, this study is the only proposal to date that achieves a satisfactory parameter estimation in critical scenarios reported in the literature [5].

## Material and methods

### The standard IRT model

The item characteristic curve of the Rash model is given by Eq 1 [1]:

$$P(Y_{i\alpha }=1|\theta_{\alpha },b_{i})=\frac{e^{\theta_{\alpha }-b_{i}}}{1+e^{\theta_{\alpha }-b_{i}}},$$ (1)

where Y_iα_ = {0, 1} is a binary variable that indicates whether examinee α correctly answered item i; θ_α_ is the ability parameter of examinee α; b_i_ is the difficulty parameter of item i; P(Y_iα_ = 1|θ_α_, b_i_) is the probability that a randomly chosen examinee with ability θ_α_ correctly answers item i; and the probability of an incorrect response is equal to P(Y_iα_ = 0|θ_α_, b_i_) = 1 − P(Y_iα_ = 1|θ_α_, b_i_).

In the Rasch model, b_i_ represents the required ability for any examinee to have a 50% (0.50) chance of correctly answering item i. Given M examinees and V items, estimates for θ_α_ and b_i_ in the IRT models are found using the likelihood shown in Eq 2 [1]:

$$L(\vec{Y}|\vec{\theta },\vec{b})=\prod_{\alpha =1}^{M}\prod_{i=1}^{V}{\left(P(Y_{i\alpha }=1|\theta_{\alpha },b_{i})\right)}^{y_{i\alpha }}{\left(1-P(Y_{i\alpha }=1|{b_{i},\theta }_{\alpha })\right)}^{{1-y}_{i\alpha }},$$ (2)

where $\vec{\theta }=(\theta_{1},\theta_{2},\ldots ,\theta_{M})$ is the vector of the examinee abilities, $\vec{b}=(b_{1},b_{2},\ldots ,b_{V})$ is the vector of the item difficulties and $\vec{Y}$ is the vector of the observed responses. To use Bayesian estimation, the prior distributions *f*(θ_α_) and *f*(b_i_) must be defined. Since $\vec{\theta }⊥\vec{b},{\vec{\theta }}_{r}⊥{\vec{\theta }}_{s},{\vec{b}}_{p}⊥{\vec{b}}_{q}$, for r ≠ s and p ≠ q, then the joint posterior distribution for parameters $\vec{\theta }$ and $\vec{b}$ is given by Eq 3.

$$f(\vec{\theta },\vec{b}\left|\vec{y})\propto L(\vec{y}\right|\vec{\theta },\vec{b})\prod_{i=1}^{V}f(b_{i})\prod_{\alpha =1}^{M}f(\theta_{\alpha }).$$ (3)

Furthermore, the ability and item parameters cannot be estimated simultaneously using maximum likelihood because of the scale indeterminacy [1, 23]. This problem can be solved by choosing arbitrary scales for either the ability or the item parameters. Using classic (frequentist) statistical analysis, the mean parameter of the abilities is usually set to zero. Using Bayesian modelling, the scale constraint can be imposed by means of the prior distribution of $\vec{\theta }$. Thus, each latent ability θ_α_ is assumed to come from a standard normal distribution. Further information regarding Bayesian estimation of IRT models can be found in [23]. A standard prior distribution for the item parameter is given by Eq 4 [23–26]:

$$b_{i}|\mu_{b},\sigma_{b}^{2}\sim N(\mu_{b},\sigma_{b}^{2}).$$ (4)

where N(μ_b_, σ_b_^2^) comprises a normal distribution with a mean of μ_b_ and a variance of σ_b_^2^. One can use Markov chain Monte Carlo (MCMC) methods to sample from the posterior distribution of μ_b_ and σ_b_^2^. Details about MCMC in the context of IRT are found in [25]. In S1 Table, a BUGS (Bayesian inference Using Gibbs Sampling) [27] code for the adjustment of the standard Rasch model is found.

### Inference under examinee choice design

When allowing examinee choice, the likelihood equation includes the missing data indicator vector $\vec{R}=(R_{1,1},R_{1,2},\ldots ,R_{i,\alpha },\ldots ,R_{V,M})$, where R_iα_ = 1 if the response of examinee α for item i is observed; otherwise, R_iα_ = 0. Given $\vec{R}$, the set of responses $\vec{Y}$ can be written as $\vec{Y}=({\vec{Y}}_{obs},{\vec{Y}}_{mis})$ [28], where ${\vec{Y}}_{obs}$ denotes the observed values and ${\vec{Y}}_{mis}$ denotes the missing values. Thus, the likelihood function given in Eq 2 is rewritten [5] as shown in Eq 5:

$$L\left[\left({\vec{\text{Y}}}_{obs},{\vec{\text{Y}}}_{mis}\right),\vec{R}|\vec{\theta },\vec{b})\right]=f[\vec{R}|\left({\vec{\text{Y}}}_{obs},{\vec{\text{Y}}}_{mis}\right),\vec{\theta },\vec{b}]\;f\left[\left({\vec{\text{Y}}}_{obs},{\vec{\text{Y}}}_{mis}\right)|\vec{\theta },\vec{b}\right],$$ (5)

and the joint posterior distribution is given by Eq 6:

$$f(\vec{\theta },\vec{b},{\vec{Y}}_{miss}|\vec{R},{\vec{Y}}_{obs})\propto f\left[\vec{R}|{\vec{\text{Y}}}_{obs},{\vec{\text{Y}}}_{mis},\vec{\theta },\vec{b}\right]\;f\left[{\vec{\text{Y}}}_{obs},{\vec{\text{Y}}}_{mis}|\vec{\theta },\vec{b}\right]\prod_{i=1}^{V}f(b_{i})\prod_{\alpha =1}^{M}f(\theta_{\alpha }).$$ (6)

Bradlow and Thomas [5] showed that if examinees are allowed to choose items, then valid statistical inference for $\vec{\theta }$ and $\vec{b}$ can be obtained using Eq 2 only if the assumptions in Eqs 7 and 8 hold:

$$f\left[\vec{R}|\left({\vec{\text{Y}}}_{obs},{\vec{\text{Y}}}_{mis}\right),\vec{\theta },\vec{b}\right]=f\left[\vec{R}|{\vec{\text{Y}}}_{obs},\vec{\theta },\vec{b}\right],$$ (7)

$$f\left[\vec{R}|{\vec{\text{Y}}}_{obs},\vec{\theta },\vec{b}\right]=f\left[\vec{R}|{\vec{\text{Y}}}_{obs}\right].$$ (8)

Assumption (7) is known as the missing at random (MAR) assumption and implies that examinees are not able to distinguish which items they would likely answer correctly. Assumption (8) implies that examinees of different abilities generally do not broadly select easier or more difficult items. Further details are found in [5]. If both assumptions (7) and (8) hold, then the posterior distribution can be rewritten as shown in Eq 9:

$$f\left(\vec{\theta },\vec{b},{\vec{Y}}_{miss}|\vec{R},{\vec{Y}}_{obs}\right)\propto f\left({\vec{Y}}_{obs}|\vec{\theta },\vec{b}\right)f\left({\vec{Y}}_{miss}|\vec{\theta },\vec{b}\right)\prod_{i=1}^{V}f(b_{i})\prod_{\alpha =1}^{M}f(\theta_{\alpha })$$ (9)

In this case, it is assumed that the process that generates missing data is non-informative. Details about statistical inference in the presence of missing data are found in [28]. In Bayesian inference, ${\vec{Y}}_{miss}$ can be treated as a random quantity (details in [28]); thus, it can be sampled from the posterior distribution [25]. This sampling is possible because Eq 5 is an augmented data likelihood. Hereafter, it is assumed that if the examinees are randomly selecting items, then the unobserved values are MAR; otherwise, the process that generates missing data is informative, and the unobserved values are not MAR.

Wang, Wainer and Thissen [4] conducted an experiment called “choose one, answer all” to test whether the MAR assumption is empirically plausible. In the experiment, 225 students indicated which items in pairs of questions they would rather answer. However, they still had to answer all items. The results showed that the MAR assumption did not hold. For example, a particular pair of items (items 11 and 12) was introduced to the students. One item (item 12) was much more difficult than the other item (item 11). It was observed that only 20% of the examinees preferred to answer item 12. It was further observed that those students who had chosen item 12 performed far better on item 11, which indicated that they had made a disadvantaged choice. Moreover, the examinees who chose item 11 performed better on both items than those who chose item 12. These results were observed elsewhere [29, 30], suggesting that students with higher abilities are more likely to differentiate difficulties between items.

Furthermore, Bradlow and Thomas [5] performed simulation studies to demonstrate the bias in the estimated parameter using the standard Rasch model in violation of assumptions (7) and (8). The complete data set had 5,000 examinees and 200 items. In the simulation study, 50 items were mandatory, and the remaining 150 items were divided into 75 choice pairs. In the experiment in which the first assumption (7) was violated, there occurred a consistent underestimation of item difficulty for the 50 mandatory items and more severe underestimation for the remaining 75 choice pairs. Furthermore, in the experiment in which the second assumption (8) was violated, there occurred overestimation of item difficulty for high-difficulty items and underestimation for low-difficulty items. The authors also stated that very little is known about the nature and magnitude of realistic violations of those assumptions.

Wang et al. [8] proposed the inclusion of a random effect parameter γ_α_ in the standard IRT models to account for the choice effect, as shown in Eq 10:

$$P(Y_{i\alpha }=1|\theta_{\alpha },\gamma_{\alpha },b_{i})=\frac{e^{\theta_{\alpha }+\gamma_{\alpha }-b_{i}}}{1+e^{\theta_{\alpha }+\gamma_{\alpha }-b_{i}}}.$$ (10)

Eq 10 is similar to Eq 1 with the inclusion of the choice effect parameter γ_α_. In this model, the ability parameter θ_α_ is separated from the choice effect, and therefore, the abilities can be compared across different examinees. In this case, θ and γ follow a bivariate normal distribution. Supposedly, the inclusion of the choice parameter γ_α_ would make assumptions (7) and (8) valid. The proposed model produced better results than the standard IRT model in some simulated scenarios. Nevertheless, the authors state that if assumption (7) or assumption (8) is violated, as described in [5], valid statistical inferences are not obtained using the standard IRT model or the proposed model with the choice effect parameter.

Liu and Wang [31] also proposed a two-dimensional IRT model that includes a choice effect parameter γ_α_. The model accounts for the random selection of items using a nominal response model [32]. For example, let a test be composed of *V* items from which examinees are free to choose *v* items (*v* < *V*). The number of possible choices, denoted by W, is equal to the number of different combinations of choosing *v* of the *V* items, i.e., $W=\left(\begin{array}{c} V\\ v \end{array}\right)$. Let $M_{\alpha }$ be the index of the item choice combination for examinee α, $M_{\alpha }\in \{1,\ldots ,k,\ldots ,W\}$. According to the nominal response model, the probability of examinee α selecting the k^th^ choice combination is defined as:

$$P(M_{\alpha }=k|\gamma_{\alpha },q_{k},p_{k})=\frac{exp(q_{k}\gamma_{\alpha }+p_{k})}{\sum_{i=1}^{W}exp(q_{i}\gamma_{\alpha }+p_{i})}$$ (11)

where $q_{k}$ is the slope parameter and $p_{k}$ is the intercept parameter for choice k. The proposed model uses the vector of choice combinations $\vec{M}=(M_{1},M_{2},\ldots .,M_{\alpha },\ldots M_{M})$ rather than the missing data indicator vector $\vec{R}$. In this case, the joint distribution for the complete data is given by Eq 12:

$$f({\vec{Y}}_{obs},{\vec{Y}}_{miss},\vec{M},\vec{\theta },\vec{\gamma },\vec{b},\vec{\varsigma })\propto f(\vec{M}|\vec{\gamma },\vec{\varsigma })f({\vec{Y}}_{obs},{\vec{Y}}_{miss}|\vec{\theta },\vec{b})f(\vec{b})f(\vec{\varsigma })f(\vec{\theta },\vec{\gamma })$$ (12)

where $\vec{\varsigma }=\{\vec{q},\vec{p}$} is the set of parameters defined in Eq 11. Furthermore, the $\vec{\theta }$ and $\vec{\gamma }$ vectors can be correlated. The probability density function (PDF) $f(\vec{M}|\vec{\gamma },\vec{\varsigma })$ is known as the missingness distribution, and vector $\vec{M}$ is assumed to be conditionally independent of ${\vec{Y}}_{obs},{\vec{Y}}_{miss},\vec{\theta }$ and $\vec{b}$, given $\vec{\gamma }$ and $\vec{\varsigma }$. The PDF $f({\vec{Y}}_{obs},{\vec{Y}}_{miss}|\vec{\theta },\vec{b})$ represents the standard IRT model given by Eq 1. The authors performed two simulation studies in which the proposed model yielded satisfactory parameter recovery. Nevertheless, the authors stated that the two simulation studies were rather simple and that the findings may not be generalizable to more complex situations.

The model proposed by Liu and Wang [31] has a limitation. The estimates of the model are possible only if the number of possible choices (W) is relatively small. The authors indicate that the number of examinees should be at least 10 times the number of item parameters in order to achieve reliable estimates in the nominal response model equation. As will be shown, this paper evaluates a scenario in which examinees are allowed to choose 20 items from a total of 50 items. This scenario requires a nominal response model with $2\times \left(\begin{array}{c} 50\\ 20 \end{array}\right)=9.425842\times 10^{13}$ item parameters. Consequently, the minimum number of examinees required to estimate the model is 9.425842 × 10^14^, which is impractical.

### Network information

Pena and Costa [33] proposed a novel representation of examinees and their selected items using network analysis. The data set was coded as layers, vertices (or nodes) and edges. Briefly, a network (or graph) $G=(\vec{V},\vec{E})$ consists of a set of *V* vertices ($\vec{V}$) that identify elements of a system and a set of E edges $\left(\vec{E}\right)$ that connect pairs of vertices {*v*_*i*_, *v*_*j*_} {*v*_*i*_, *v*_*j*_}, pointing out the presence of a relationship between the vertices [34]. In the proposed network representation, each examinee is represented as a single network in which the *V* items are the vertices and every pair of the *V*_*α*_ selected items is connected by an edge. That is, the data set is initially represented as M single-layer networks or a multilayer network $\vec{G}=(G_{1},G_{2},\ldots ,G_{M})$ [35]. From the multilayer network, two matrices are created.

The first matrix, called the overlapping matrix, **O** = [*o*_ij_], is a weighted *V* × *V* matrix in which the elements *o*_ij_ indicate the number of examinees that chose both items i and j [35, 36]:

$$o_{ij}=\sum_{\alpha =1}^{M}a_{ij}^{\left[\alpha \right]}$$ (13)

where *ɑ*_ij_^[α]^ = 1 if examinee α chose both items i and j and = 0 otherwise; 0 ≤ *o*_ij_ ≤ M,∀i, j. The second matrix, called matrix **U** = [*u*_ij_], is a binary *V* × *V* matrix in which *u*_ij_ is equal to 1 if *o*_ij_ (Eq 13) is greater than a threshold (Ψ) and zero otherwise. The threshold is calculated to identify recurrent edges in the multilayer network and is given by Eq 14:

$$\Psi =\frac{E^{\left[o\right]}}{\eta_{\varepsilon }}+Z_{\gamma }\sqrt{\frac{E^{\left[o\right]}}{\eta_{\varepsilon }}(1-\frac{1}{\eta_{\varepsilon }})},$$ (14)

where $E^{\left[o\right]}=\sum_{\alpha =1}^{M}\frac{V_{\alpha }(V_{\alpha }-1)}{2}$ is the total number of edges in the multilayer network, $\eta_{\varepsilon }=\frac{V(V-1)}{2}$ is the maximum number of edges in a single-layer network and *Z*_γ_ is a z-score statistic (*Z*_0,05_ = 1.645). Under the null hypothesis, the statistical distribution of the number of incident edges in each pair of vertices is the same. Thus, Ψ represents the upper bound of the observed number of edges between vertices iand j under the hypothesis that the E^[o]^ edges are randomly distributed. Further details are found in [33]. Therefore, matrix **U** is a binary matrix that preserves only the statistically significant edges, as shown in Eq 15:

$$u_{ij}=\{\begin{array}{cc} 1, & if\;o_{ij}>\Psi ;\;i\neq j;\\ 0, & otherwise. \end{array}$$ (15)

In [33], it was shown that the density of matrix **U** can be used to test whether the MAR assumption holds. The larger the density of matrix **U** is, the more violated the MAR assumption; that is, the density of matrix **U** indicates the violation level of the MAR assumption. The density of a network $G=(\vec{V},\vec{E})$ is given in Eq 16 [37]:

$$D=\frac{2E}{V(V-1)}.$$ (16)

Moreover, in [33], several network centrality measures and their correlations with item difficulty when the MAR assumption is violated were evaluated. The most frequent centrality measures found in the literature [38] were tested. The eigenvector of matrix **O** was found to be the most consistent and robust network statistic to estimate item difficulty. The eigenvector centrality of a vertex i (ρ_i_) is the *i*-*th* element of the first eigenvector of matrix **O**:

$$\lambda \vec{\rho }=Ο\vec{\rho }$$ (17)

where λ is the eigenvalue and $\vec{\rho }$ is the first eigenvector of matrix **O**. Further details about eigenvector centrality are found in [39]. In addition, the simulation study presented in [33] indicates that the larger the MAR assumption violation is, the larger the correlation between the eigenvector centrality and item difficulties. It is worth mentioning that the eigenvector centrality assumes values within the range 0–1. Therefore, it provides a standardized measure of vertex centrality.

### The proposed model

In general, the relation between item difficulty b_i_ and the first eigenvector of matrix **O** can be written as shown in Eq 18.

$$b_{i}=g(\rho_{i})$$ (18)

where *g*(ρ_i_) is a function of the *i*-*th* element of the first eigenvector $\vec{\rho }$. This paper proposes a new IRT model that takes into account the relation shown in Eq 18. This can be achieved defining the following prior distribution:

$$b_{i}|\mu_{b_{i}},\sigma_{b}^{2}\sim N(\mu_{b_{i}},\sigma_{b}^{2}),$$ (19)

where $\mu_{b_{i}}=g(\rho_{i})$ and σ_b_^2^ accounts for the variability of b_i_ that cannot be explained by *g*(ρ_i_). It is worth mentioning that the larger the correlation between $\vec{\rho }$ and $\vec{b}$ is, the lower the dispersion parameter σ_b_^2^. The posterior distribution, shown in Eq 6, can be rewritten as shown in Eq 20:

$$\begin{array}{l} f\left(\vec{\theta },\vec{b},{\vec{Y}}_{miss}|\vec{\rho },\vec{R},{\vec{Y}}_{obs}\right)\propto f\left[\vec{R}|\vec{\rho },{\vec{\text{Y}}}_{obs},{\vec{\text{Y}}}_{mis},\vec{\theta },\vec{b}\right]\\ \;\times f\left[{\vec{\text{Y}}}_{obs},{\vec{\text{Y}}}_{mis}|\vec{\rho },\vec{\theta },\vec{b}\right]\prod_{i=1}^{V}f(b_{i}|\rho_{i})\;\prod_{\alpha =1}^{M}f(\theta_{\alpha }). \end{array}$$ (20)

where

$$f\left[\vec{R}|\vec{\rho },{\vec{\text{Y}}}_{obs},{\vec{\text{Y}}}_{mis},\vec{\theta },\vec{b}\right]\propto f\left[\vec{R}|\vec{\rho },{\vec{\text{Y}}}_{obs}\right].$$ (21)

Eq 21 assumes that, given $\vec{\rho }$, the missing data indicator $\vec{R}$ is independent of ${\vec{Y}}_{mis},\vec{\theta }$ and $\vec{b}$. Further information about the conditioning on covariates for the missingness mechanism becoming negligible are found in [40, 41].

In this paper, the following mathematical model between $\vec{\rho }$ and $\vec{b}$ is proposed:

$$b_{i}=-\frac{1}{\beta_{1}}(\text{log}(\frac{1-(\rho_{i}-\beta_{2})}{(\rho_{i}+\beta_{2})-C})+\beta_{0})$$ (22)

where β_0_, β_1_ and β_2_ are coefficients to be estimated and C is the minimum value of ρ_i_, i.e., $C=\text{min}\left(\vec{\rho }\right).$ This model represents the inverse equation of a logistic function with a shape that asymptotically tends to the lowest value of $\vec{\rho }$. The β_2_ parameter is required to move the vector $\vec{\rho }$ below 1. Furthermore, the β_2_ parameter is also used to shift vector $\vec{\rho }$ above its lowest value to prevent $\text{log}(\frac{1-(\rho_{i}-\beta_{2})}{0})=+\infty$. This model was empirically proposed based on simulation studies, as shown in the Results section. It is worth mentioning that other functions to describe the relation between $\vec{\rho }$ and $\vec{b}$ can be proposed. The BUGS code for the proposed model is available in S2 Table.

### Simulation study

To investigate the data behaviour under the violations of assumptions (7) and (8), we performed several simulations using three different scenarios. In all scenarios, 1,000 examinees (α = 1,…, 1,000) choose 20 items within a total of 50 items (i = 1, …, 50). The examinee ability (θ_α_) and item difficulty (b_i_) were both generated from a standard normal density distribution and held fixed. The complete data set can be represented by a 1,000 versus 50 dimensional matrix of binary responses. For each examinee α and item i, the probability of a correct answer was calculated using Eq 1. This probability was used to generate the outcome Y_iα_ using a Bernoulli random number generator.

In the first scenario, hereafter named scenario 1, both assumptions (7) and (8) were valid. That is, the items were randomly selected by the examinees, and consequently, the process that causes missing data was non-informative. Therefore, this is the scenario in which valid statistical inference can be obtained using the standard Rasch model.

The second scenario, named scenario 2, is identical to the first simulation scenario presented in [33]. In this scenario, each examinee chooses items based on current values of θ_α_ and b_i_. That is, for each examinee α, the items are divided into two groups: the first group comprises items that are easier than the examinee ability, i.e., b_i_ ≤ θ_α_. This is the group in which the examinee has a probability of more than 0.50 to achieve a correct answer. The second group comprises items that are more difficult than the examinee ability, i.e., b_i_ > θ_α_. In this group, the examinee has a probability of less than 0.50 to answer the items correctly. A weight value (w_i_) is assigned to the items in each group. For items in group 2, the weight value is w_i_^[2]^ = 1; for items in group 1, the weight value varies from 1.5 to 30: w_i_^[1]^ ∈ {1.5, 2, 5, 10, 30}. For example, if w_i_^[1]^ = 2, then it can be said that the items in group 1 have double the chance of being selected by the examinee than the items in group 2. In this scenario, assumptions (7) and (8) are violated.

Finally, in the third scenario, named scenario 3, the examinee choice depends on y_*mis*_. That is, assumption (7) is violated. Similar to scenario 2, for each examinee α the items are divided into two groups. In scenario 3, the first group comprises items that were correctly answered by the examinee in the complete data set (y_iα_ = 1), and the second group contains the items that the examinee failed (y_iα_ = 0). Similar to scenario 2, for items in group 2, the weight value is w_i_^[2]^ = 1; for items in group 1, the weight value varies from 1.5 to 30: w_i_^[1]^ ∈ {1.5, 2, 5, 10, 30}. This scenario is similar to the second simulation study described in [5].

Table 1 summarizes the proposed simulations. Each configuration was replicated 100 times.

**Table 1:** Summary of the proposed simulations.

Scenario	Manipulated factors		Number of replications
	Group of items	Weights	
**1**	One group	The same for all items w_i_ = 1	100
**2**	Group 1: b_i_ ≤ θ_α_ Group 2: b_i_ > θ_α_	w_i_^[1]^ = 1.5 and w_i_^[2]^ = 1	100
		w_i_^[1]^ = 2 and w_i_^[2]^ = 1	100
		w_i_^[1]^ = 5 and w_i_^[2]^ = 1	100
		w_i_^[1]^ = 10 and w_i_^[2]^ = 1	100
		w_i_^[1]^ = 30 and w_i_^[2]^ = 1	100
**3**	Group 1: y_iα_ = 1 Group 2: y_iα_ = 0	w_i_^[1]^ = 1.5 and w_i_^[2]^ = 1	100
		w_i_^[1]^ = 2 and w_i_^[2]^ = 1	100
		w_i_^[1]^ = 5 and w_i_^[2]^ = 1	100
		w_i_^[1]^ = 10 and w_i_^[2]^ = 1	100
		w_i_^[1]^ = 30 and w_i_^[2]^ = 1	100

It is worth mentioning that the selected items were generated using a multinomial probability distribution. That is, the probability of examinee α selecting item i is:

$$p_{i\alpha }=\frac{w_{i\alpha }}{\sum_{i}w_{i\alpha }}.$$ (23)

In scenario 1, w_iα_ = 1 ∀ i.

## Results and discussion

In this section, we first present empirical evidence of the proposed function, given in Eq 22, to describe the relation between $\vec{b}$ and $\vec{\rho }$. Second, we define values for the variance parameter of the prior distribution (σ_b_^2^). Prior values of the σ_b_^2^ parameter improve the statistical properties of the proposed model. Finally, several simulation studies are performed in different conditions to compare the accuracy of parameter recovery obtained using the standard Rasch model and using our proposed model.

### Empirical validation of the proposed model

To evaluate the performance of the proposed function (Eq 22) to predict item difficulty, using the first eigenvector of matrix **O**, we performed 10,000 Monte Carlo simulations for scenarios 2 and 3 and calculated the residual sum of squares (RSS), as shown in Eq 24.

$$RSS=\sum_{i=i}^{V}{\left(b_{i}-{\hat{b}}_{i}\right)}^{2}$$ (24)

where ${\hat{b}}_{i}$ is the fitted value of b_i_ using Eq 22. It is worth mentioning that the smaller the value of RSS is, the better the fit of the model and the lower the bias of the estimates. The β_0_, β_1_ and β_2_ coefficients were estimated using the least squares method adapted to a nonlinear model in the software R [42, 43]. Table 2 shows the minimum, mean, maximum and standard deviation values of the RSS for different weight values used in scenarios 2 and 3. In both scenarios, the larger the weights are, the lower the RSS. Thus, there is empirical evidence that the proposed function seems to provide a better fit in situations in which the MAR assumption is more violated.

**Table 2:** Summary of the residual sum of squares.

Weight	Scenario 2				Scenario 3			
	Min	Mean	Max	Sd	Min	Mean	Max	Sd
**1.5**	4.6521	10.0364	20.5177	1.8479	7.7194	16.793	32.3424	3.3918
**2**	2.1942	5.8299	11.0088	1.2245	2.9474	7.4835	13.6649	1.5131
**5**	0.6577	2.5018	5.5351	0.825	0.7303	1.9269	4.2557	0.4335
**10**	0.4425	1.9922	4.6373	0.7541	0.4524	1.1196	2.4593	0.2501
**30**	0.2682	1.8019	4.4424	0.7522	0.2799	0.738	1.5916	0.1545

**Fig 1:**
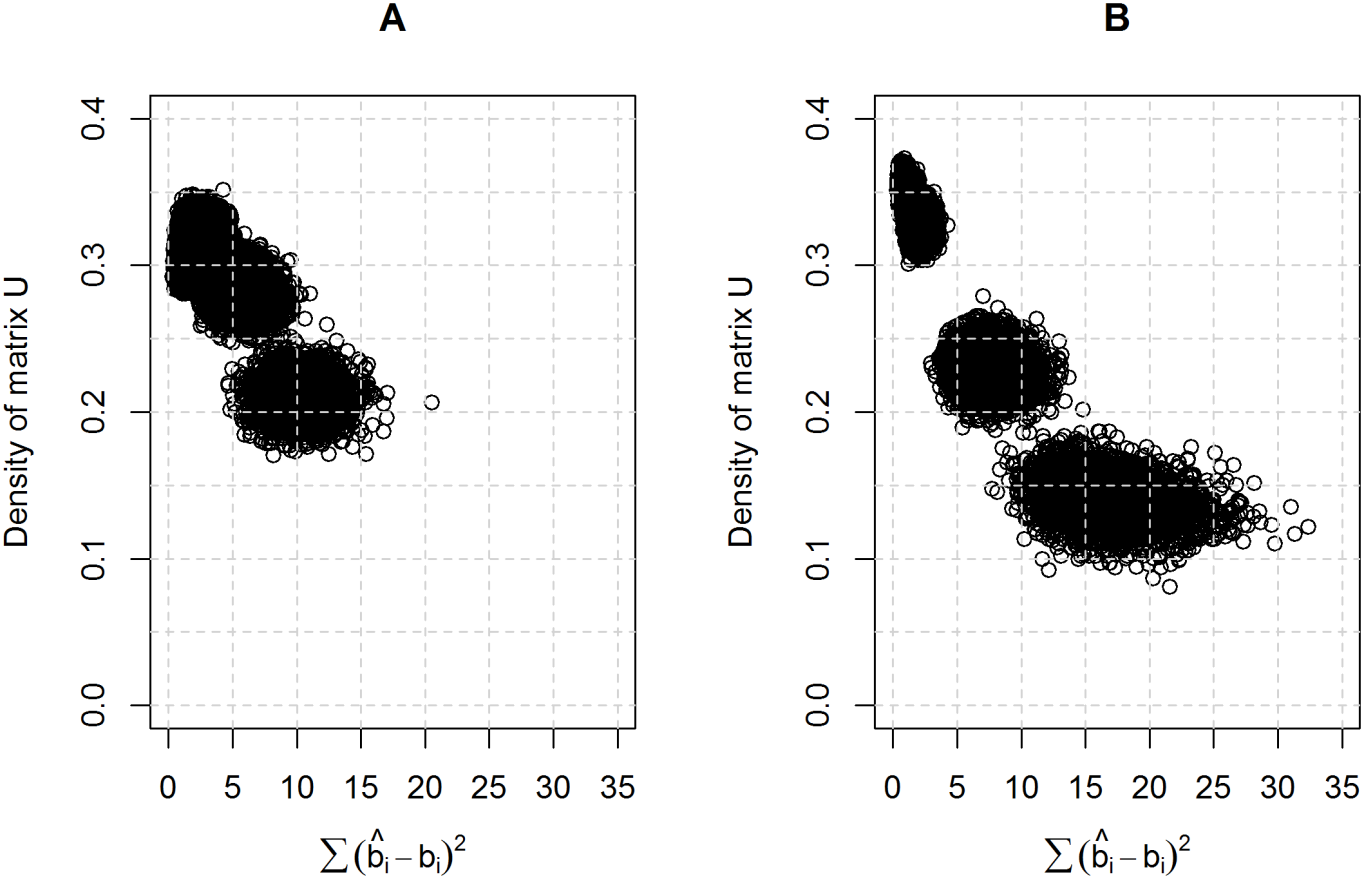
Residual sum of squares versus the density of matrix U. (A) Scenario 2. (B) Scenario 3.

Fig 1A shows the density of matrix **U** versus the RSS, using data generated from scenario 2. Fig 1B shows the density of matrix **U** versus the RSS, using data generated from scenario 3. In general, the larger the density of matrix **U** is, the lower the RSS. In [33], it was shown that the larger the density of matrix **U** is, the stronger the MAR assumption violation. Therefore, the density of matrix **U** can be used as a predictor of the goodness-of-fit statistic of the proposed model, as shown in Eq 22.

We propose using the observed density value of matrix **U** to choose different values for σ_b_^2^ in the prior distribution (Eq 19). Lower prior values for σ_b_^2^ mean that the posterior distribution of b_i_ will be concentrated towards its mean, $\mu_{b_{i}}=g(\rho_{i})$, i.e., the posterior estimate of b_i_ is mostly defined by the proposed eigenvector centrality function. In contrast, the larger the prior value of σ_b_^2^ is, the less the posterior estimate of b_i_ is affected by the eigenvector centrality function.

Based on the results shown in Fig 1 and further simulation studies, the proposed values of σ_b_^2^ are shown in Table 3. As previously mentioned, the lower the density of matrix **U** is, the larger the prior value of σ_b_^2^. Furthermore, if the density of matrix **U** is within 0.0–0.1, there is empirical evidence that the MAR assumptions holds. In this case, a large variance value is chosen in order to make the prior distribution for b_i_ less informative. Future work includes exhaustive simulation studies to propose a mathematical function that relates the density of matrix **U** and the prior value of σ_b_^2^, thus further minimizing the bias of the proposed model.

**Table 3:** The proposed values for the variance of the prior distribution.

Density of matrix U	Proposed value for σ_b_^2^
[0–0.10]	10
(0.10–0.20]	5
(0.20–0.25]	0.5
(0.25–0.30]	0.4
(0.30–0.35]	0.3
> = 0.35	0.2

**Fig 2:**
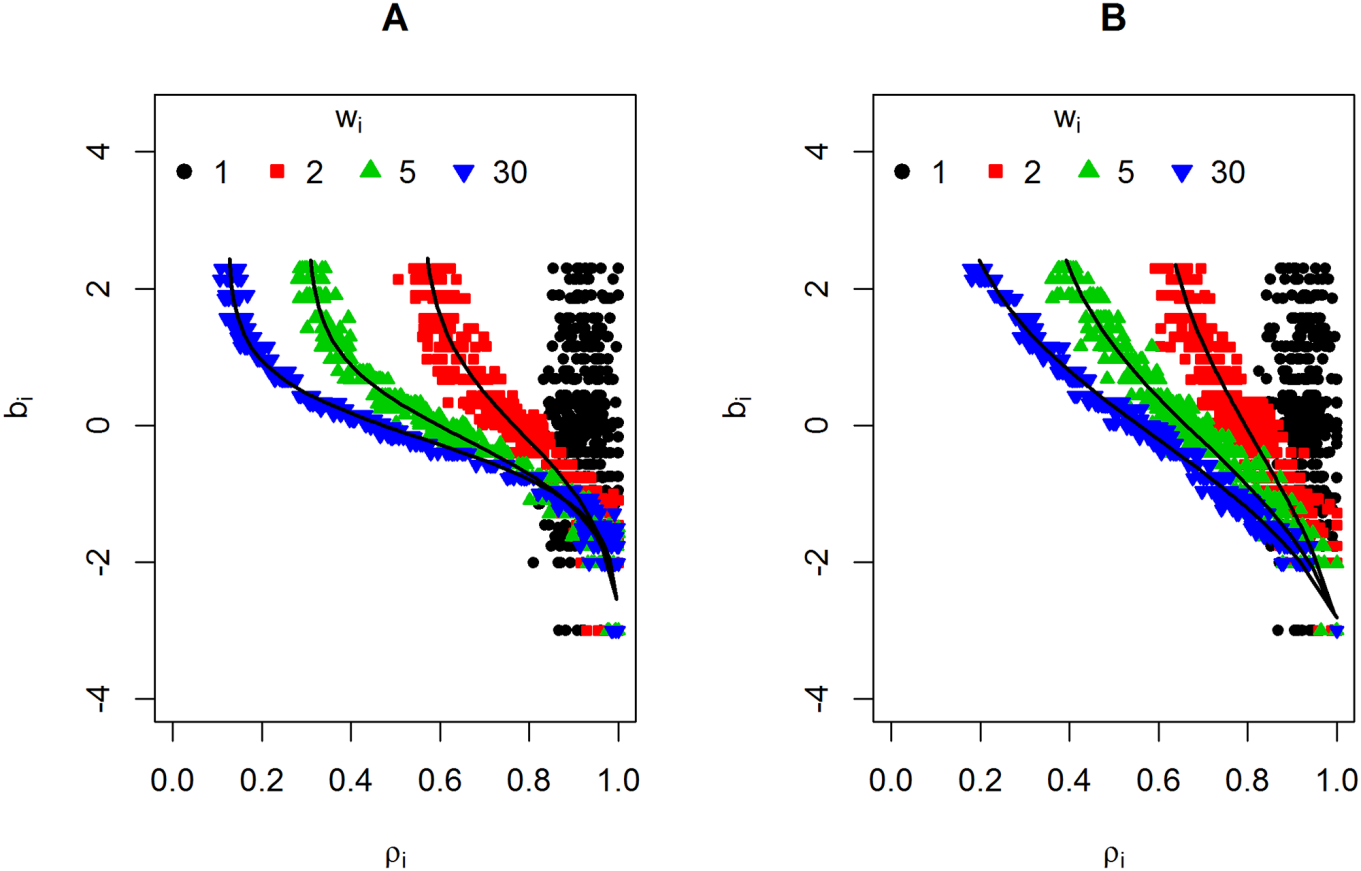
Item difficulty versus the first eigenvector of matrix O. (A) Scenario 2. (B) Scenario 3. The black lines represent the proposed function (Eq 22).

Fig 2 illustrates the proposed function. It shows the plot of b_i_ versus ρ_i_ for 10 Monte Carlo simulations. The black circles represent the data generated from scenario 1 (w_i_ = 1) in which there is no correlation between $\vec{b}$ and $\vec{\rho }$. With the exception of the black circles, Fig 2A shows the data generated from scenario 2, and Fig 2B shows the data generated from scenario 3 using three different weight values, w_i_ ∈ {2, 5, 30}. The proposed function was adjusted using the mean value of ρ_i_ for each simulated weight, that is, ${\overset{-}{\rho }}_{i}=\sum_{i=1}^{10}\rho_{i}$, and is represented with black lines. In both scenarios, the proposed function achieved a good data fit.

### Accuracy of the estimated parameters

To evaluate the performance of the proposed model compared to the standard Rasch model, 100 Monte Carlo simulations were performed under the three scenarios previously described. Scenario 1 is used as the reference scenario since in this scenario, valid statistical inferences can be obtained using the standard Rasch model [5]. The accuracy of the estimated parameters is evaluated using the bias, the square root of the mean square error (RMSE) and the square root of the maximum square error (RSE_MAX_), which are given by Eqs 25, 26 and 27, respectively.

$$Bias\left(b_{i}\right)=\sum_{n=1}^{100}({\hat{b}}_{ni}-b_{i})/100$$ (25)

$$RMSE\left(b_{i}\right)=\sqrt{\frac{1}{100}\times \sum_{n=1}^{100}{\left({\hat{b}}_{ni}-b_{i}\right)}^{2}}$$ (26)

$$RSE_{MAX}\left(b_{i}\right)=\sqrt{Max\;\left(\cup_{n=1}^{100}\left\{{\left({\hat{b}}_{ni}-b_{i}\right)}^{2}\right\}\right)}$$ (27)

where b_i_ is the true difficulty of item i and ${\hat{b}}_{ni}$ is the estimated value of b_i_ in the *n-th* Monte Carlo simulation. In addition, 95% highest probability density (HPD) intervals [44] are provided for each estimated difficulty. Using the HPD intervals, the proportion of simulated parameters that fall within the HPD intervals are calculated. It is worth mentioning that the posterior mean, i.e., the mean of the posterior distribution, was used as point estimate of b_i_ [45].

The standard Rasch model and the proposed model were estimated using the BUGS codes shown in S1 and S2 Tables. These BUGS codes can be run using one of the following free software programs: WinBugs [46], OpenBugs [47] and JAGS [48]. The WinBugs [46] software was used. The MCMC simulations were applied using one chain, 10,000 iterations, a burn-in period of 1,000 iterations and a sample period (thin) of 5 iterations. The variance of the prior distribution was set as 10 for the standard Rasch model and was selected according to Table 3 for the proposed model. The initial values of the parameters were as follows: β_0_ = 0, β_1_ = -1.5, β_2_ = 0.01, μ_b_ = 0, b_i_ = 0 ∀ i, and θ_α_ = 0 ∀α. The convergence of the model was evaluated using the autocorrelation level of the chains, which was near zero, and the trace plot, which indicated that convergence was achieved. The average time to run the standard Rasch model once was 15.10 minutes, and the average time to run the proposed model was 15.40 minutes using an Intel I Core i7 processor with 2 GHz and 8 GB of RAM. Further details about the model specifications in WinBugs can be found in [49].

**Fig 3:**
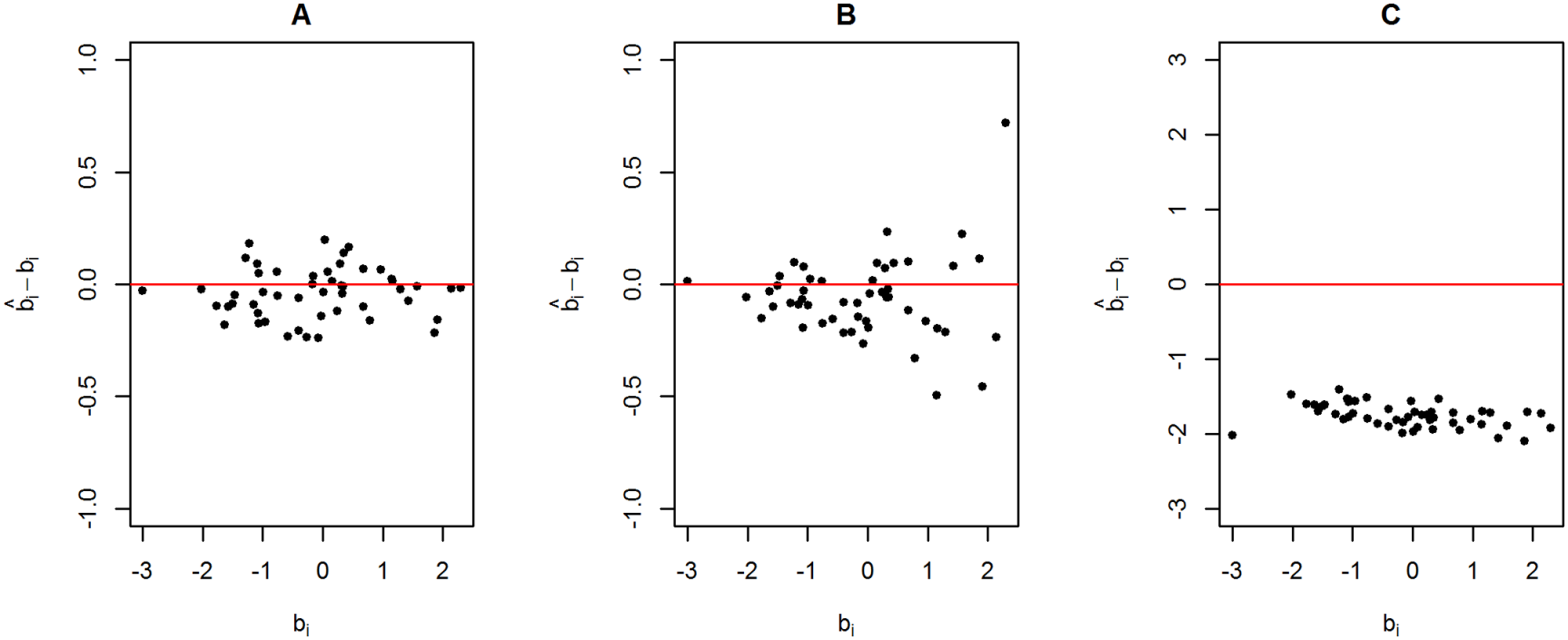
Estimates minus true item difficulties plotted against true item difficulties for three simulated data sets. (A) Data set generated from scenario 1. (B) Data set generated from scenario 2 using a weight value equal to 10. (B) Data set generated from scenario 3 using a weight value equal to 10.

**Fig 4:**
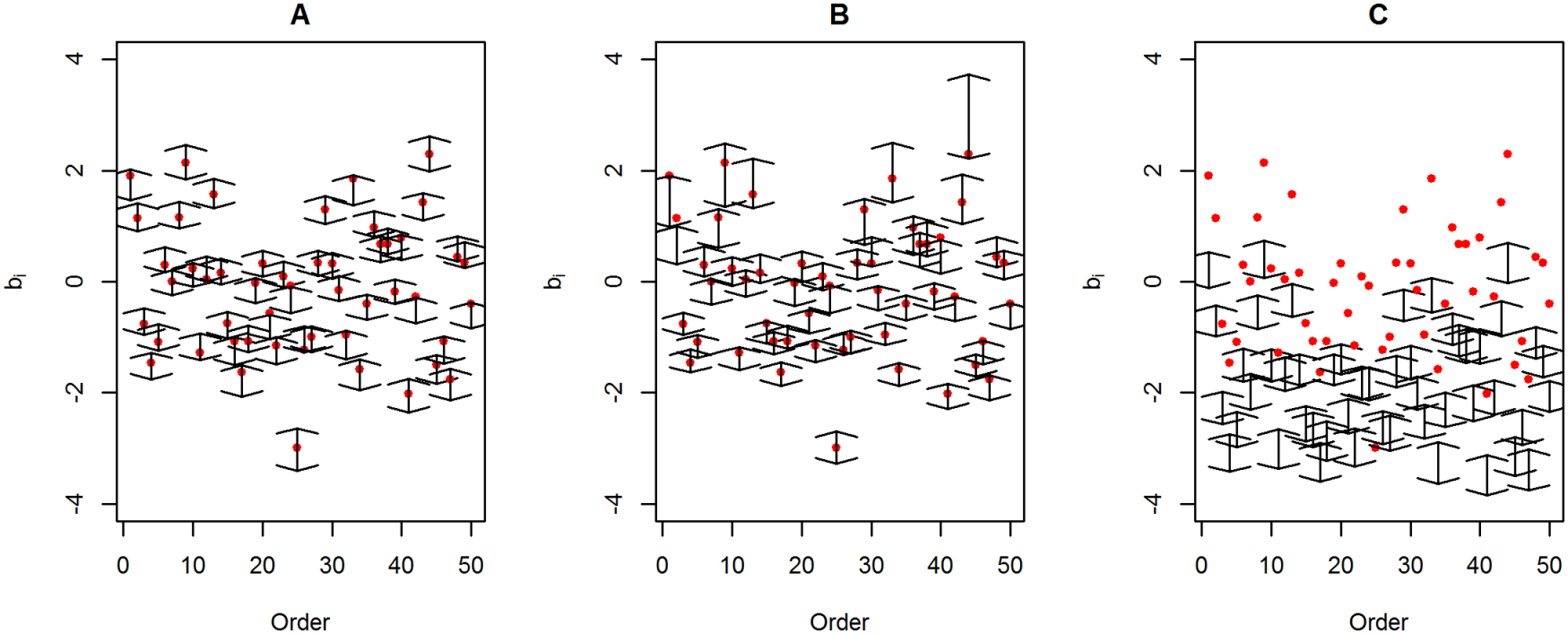
95% HPD plotted according the item order for three simulated data sets. The red points represent the true item difficulties. (A) Data set generated from scenario 1. (B) Data set generated from scenario 2 using a weight value equal to 10. (B) Data set generated from scenario 3 using a weight value equal to 10.

Fig 3 shows the differences between the estimates using the standard Rach model and the true item difficulties plotted against the true item difficulties. Fig 3A shows the results using data generated from scenario 1. Fig 3B and 3C show the results using data generated from scenario 2 and scenario 3, respectively, using a weight value equal to 10. Fig 3A shows that the residues (${\hat{b}}_{i}-b_{i}$) behave as a random cloud around zero. In contrast, Fig 3B shows that the dispersion of the residues around zero is larger for more difficult items. Fig 3C shows a severe underestimation of the item difficulties. Fig 4 shows the estimated 95% HPD intervals and the true item difficulties (red points). The HPD obtained using the data set generated from scenario 1 (Fig 4A) and scenario 2 (Fig 4B) contains most of the true item difficulties. It is worth noting that some of the HPD intervals shown in Fig 4B are larger than those in Fig 4A. In contrast, none of the HPD intervals shown in Fig 4C (scenario 3) contain the true item difficulties, which shows a serious model fitting problem.

The empirical results showed that the fitting problem reported in scenario 3 cannot be solved by replacing the standard Rasch model with the proposed model. It can be seen that the variability of the bias is smaller than in scenario 2; nevertheless, the estimated difficulties are consistently shifted towards lower values. We suggest fixing this problem by fitting the proposed model in two stages. In the first stage, the examinees are free to choose *v* within V items. In the second stage, a few of those V items are set as mandatory (*v*_*c*_), and the examinees are required to answer these *v*_*c*_ items again. Thus, the *v*_*c*_ items will be estimated twice as follows: in the first stage, the *v*_*c*_ items are adjusted using our proposed method; in the second stage, the complete responses of the *v*_*c*_ items are adjusted separately using the standard IRT model. The average difference between the estimated difficulties in the second and the first stages of the *v*_*c*_ items is calculated and added to the first difficulty estimates of all V items. This procedure adds a constant value to the estimates obtained in the first stage, thus correcting the bias shift in scenario 3. An alternative to the second stage adjustment is simply to include in the questionnaire *v*_*c*_ pre-tested items, i.e., items in which the parameter b_i_ is known in advance. In this case, the average difference will be calculated using the pre-calibrated value of b_i_ of the *v*_*c*_ items. In this case, mandatory items are not required.

In our simulation study, two items were set as mandatory to perform this calibration. The results show that two items were sufficient to correct the bias shift. Future work includes more exhaustive simulation studies to define the number of *v*_*c*_ items in the second stage. It is worth mentioning that this procedure is not necessary in scenario 2 since the residues are already centered at zero. Nevertheless, in a real situation, is it not possible to identify whether the choice was made following scenario 2 or scenario 3. Nonetheless, this procedure can be applied in both scenarios.

Table 4 shows the parameter accuracy for scenario 1 using the standard Rasch model and the proposed model. As previously mentioned, the standard Rasch model achieves valid statistical inference in this scenario and will be used as the accuracy reference of the estimated parameters. Thus, the reference model has minimum and maximum bias of -0.2542 and 0.1648, respectively. The mean and maximum values for the RMSE are 0.1225 and 0.2814, respectively. RSE_MAX_ represents the largest absolute difference between the true and estimated difficulty for each item; the mean value is 0.3251, and the maximum value is 0.7342. The largest difference between the true and estimated items difficulties in scenario 1 is 0.7342. The average width of the 95% HPD interval is 0.4996, and the maximum width is 0.8198. Finally, on average, 95.30% of the true item difficulties are inside the 95% HPD interval, and the minimum proportion is 82%.

**Table 4:** Summary of the estimated parameters in scenario 1.

STANDARD RASCH MODEL						PROPOSED MODEL				
	BIAS	RMSE	RSE MAX	HPD WIDTH	INSIDE HPD	BIAS	RMSE	RSE MAX	HPD WIDTH	INSIDE HPD
Min	-0.2542	0.0718	0.1732	0.4480	82.0%	-0.257	0.0835	0.2134	0.4509	86.0%
Mean	-0.0248	0.1225	0.3251	0.4996	95.3%	-0.0227	0.1231	0.3111	0.5014	95.0%
Max	0.1648	0.2814	0.7342	0.8198	100.0%	0.1699	0.2831	0.5715	0.8257	100.0%
Sd	0.0755	0.0359	0.1013	0.0671	3.44%	0.0784	0.0356	0.0704	0.0675	2.72%

To test whether the proposed model fits the data generated from scenario 1, a Student’s t-test was performed to compare its results with the standard Rasch model, as suggested by one referee. The p-value obtained using the bias as the dependent variable was 0.8940, that obtained using the RMSE was 0.9343 and that obtained using the RSE_MAX_ was 0.4239. Thus, it can be concluded that, assuming a significance level of 5%, the proposed model was statistically similar to the standard Rasch model regarding the average bias, the average RMSE and the average RSE_MAX_ of the item difficulties when the MAR assumption is valid. This result was found because the variance of the prior distribution when the density of matrix **U** is close to zero is sufficiently large, which makes the prior distribution less informative. It is worth noting that the maximum value of RSE_MAX_ is lower in the proposed model (0.5715) than in the standard Rasch model (0.7342). This result occurs because even though the data sets were generated using a random selection of items, a correlation between the eigenvector and the item difficulties can eventually occur in a few simulations. This correlation may be the reason why the maximum value of RSE_MAX_ is lower in the proposed model. Table 5 shows the accuracies of the estimated parameters for scenario 2 using the standard Rasch model and the proposed model with five levels of MAR assumption violation. The standard Rasch model results are close to scenario 1 for lower weight values: w_i_ = 1.5 and w_i_ = 2. For larger levels of MAR assumption violation, w_i_ = 5, w_i_ = 10 and w_i_ = 30, the RMSE and RSE_MAX_ values seem to be different from those observed in scenario 1, especially in terms of the maximum values. In contrast, the results using the proposed model seem to be closer to those observed in scenario 1, even for larger weights. Furthermore, our proposed model presented a narrower 95% HPD width and a larger average proportion of the true item difficulties inside the HPD intervals.

**Table 5:** Summary of the parameter recovery in scenario 2.

STANDARD RASCH MODEL						PROPOSED MODEL				
	Weight = 1.5					Weight = 1.5				
	BIAS	RMSE	RSE MAX	WIDTH HPD	INSIDE HPD	BIAS	RMSE	RSE MAX	WIDTH HPD	INSIDE HPD
Min	-0.2407	0.0733	0.1857	0.4497	68.00	-0.2058	0.0733	0.1773	0.4431	66.00
Mean	-0.0223	0.1333	0.3599	0.5013	93.34	-0.0190	0.1297	0.3507	0.4898	93.66
Max	0.1528	0.2660	0.6305	0.7535	100.00	0.1507	0.2356	0.5860	0.6885	100.00
Sd	0.0750	0.0326	0.0833	0.0708	5.41	0.0720	0.0308	0.0774	0.0610	5.30
	Weight = 2					Weight = 2				
	BIAS	RMSE	RSE MAX	WIDTH HPD	INSIDE HPD	BIAS	RMSE	RSE MAX	WIDTH HPD	INSIDE HPD
Min	-0.2455	0.0809	0.1751	0.4453	74.00	-0.2028	0.0634	0.1692	0.4323	70.00
Mean	-0.0246	0.1387	0.3748	0.5054	92.22	-0.0255	0.1278	0.3273	0.4816	92.94
Max	0.1476	0.2693	0.7198	0.7734	100.00	0.1611	0.2286	0.5586	0.6848	100.00
Sd	0.0788	0.0350	0.1071	0.0830	5.12	0.0740	0.0335	0.0770	0.0644	5.08
	Weight = 5					Weight = 5				
	BIAS	RMSE	RSE MAX	WIDTH HPD	INSIDE HPD	BIAS	RMSE	RSE MAX	WIDTH HPD	INSIDE HPD
Min	-0.2168	0.0738	0.1754	0.4135	64.00	-0.1821	0.0717	0.1635	0.3963	66.00
Mean	-0.0311	0.1454	0.3941	0.5315	91.96	-0.0243	0.1224	0.3162	0.4825	94.70
Max	0.1818	0.3147	0.8963	1.0369	100.00	0.1461	0.2299	0.6164	0.7751	100.00
Sd	0.0824	0.0509	0.1536	0.1497	7.17	0.0690	0.0371	0.1000	0.1004	4.85
	Weight = 10					Weight = 10				
	BIAS	RMSE	RSE MAX	WIDTH HPD	INSIDE HPD	BIAS	RMSE	RSE MAX	WIDTH HPD	INSIDE HPD
Min	-0.2356	0.0757	0.2234	0.3970	64.00	-0.1848	0.0596	0.1602	0.3830	72.00
Mean	-0.0365	0.1467	0.3930	0.5594	92.88	-0.0297	0.1238	0.3228	0.4965	94.10
Max	0.2016	0.3485	1.1578	1.2580	100.00	0.1441	0.2544	0.6915	0.8789	100.00
Sd	0.0885	0.0636	0.1718	0.2116	6.56	0.0739	0.0481	0.1262	0.1327	4.58
	Weight = 30					Weight = 30				
	BIAS	RMSE	RSE MAX	WIDTH HPD	INSIDE HPD	BIAS	RMSE	RSE MAX	WIDTH HPD	INSIDE HPD
Min	-0.3008	0.0654	0.1551	0.3837	64.00	-0.2548	0.0591	0.1234	0.3715	72.00
Mean	-0.0321	0.1664	0.4247	0.6061	91.74	-0.0479	0.1365	0.3321	0.5260	93.44
Max	0.3197	0.5310	1.3339	1.7749	100.00	0.1224	0.3141	0.6980	1.0569	100.00
Sd	0.1213	0.1009	0.2573	0.3192	6.21	0.0878	0.0632	0.1335	0.1843	5.20

Three univariate two-way analysis of variance (ANOVA) was conducted to further investigate whether the observed differences between the two models are of statistical significance in scenario 2. The bias, RMSE and RSE_MAX_ were used as dependent variables, and the estimation method (standard Rasch model or propose model) and the weight magnitude (1.5, 2, 5, 10, 30) were used as factors. The results are shown in Table 6. The null hypothesis assumes that the mean value of the dependent variable does not change among different models and/or different weights. Assuming a significance level of 5%, it can be concluded that the proposed model is significantly different from the standard Rasch model regarding the RMSE and RSE_MAX_ in scenario 2, i.e., the p-value is smaller than 5%, thus rejecting the null hypothesis.

**Table 6:** Two-way ANOVA using data generated in scenario 2.

	Estimation method	Weight values	Interaction
**BIAS**	F(1, 490) = 0.000 (p-value = 0.994)	F(4, 490) = 0.802 (p-value = 0.524)	F(4, 490) = 0.319 (p-value = 0.866)
**RMSE**	F(1, 490) = 14.111 (**p-value = 0.0002**)	F(4, 490) = 2.305 (p-value = 0.0574)	F(4, 490) = 0.969 (p-value = 0.4239)
**RSE**_**MAX**_	F(1, 490) = 22.866 (**p-value = 0.000**)	F(4, 490) = 0.605 (p-value = 0.659)	F(4, 490) = 1.364 (p-value = 0.245)

Bold text indicates significant p-values (< 0.05).

Table 7 shows the results obtained for scenario 3. In general, the RMSE and RSE_MAX_ values for both models are larger than those values observed in scenario 2. The results of the proposed model seem to differ from those values observed in scenario 1 only for w_i_ = 30, particularly the range of the bias and the maximum value of RMSE. Nevertheless, the proposed model seems to provide better results than the standard Rasch model, especially for larger levels of MAR assumption violation (w_i_ = 5, w_i_ = 10, w_i_ = 30).

**Table 7:** Summary of the parameter recovery in scenario 3.

STANDARD RASCH MODEL						PROPOSED MODEL				
	Weight = 1.5					Weight = 1.5				
	BIAS	RMSE	RSE MAX	WIDTH HPD	INSIDE HPD	BIAS	RMSE	RSE MAX	WIDTH HPD	INSIDE HPD
Min	-0.2444	0.0811	0.1954	0.4538	68.00	-0.2370	0.0806	0.1798	0.4560	76.00
Mean	-0.0254	0.1386	0.3778	0.5066	92.70	-0.0328	0.1373	0.3640	0.5085	92.86
Max	0.1593	0.2778	0.6631	0.8751	100.00	0.1493	0.2700	0.6367	0.8722	100.00
Sd	0.0749	0.0349	0.0889	0.0717	5.59	0.0763	0.0340	0.0892	0.0718	4.72
	Weight = 2					Weight = 2				
	BIAS	RMSE	RSE MAX	WIDTH HPD	INSIDE HPD	BIAS	RMSE	RSE MAX	WIDTH HPD	INSIDE HPD
Min	-0.2286	0.0823	0.1937	0.4631	74.00	-0.2263	0.0868	0.2217	0.4522	64.00
Mean	-0.0294	0.1420	0.3764	0.5159	92.88	-0.0469	0.1412	0.3600	0.5000	91.70
Max	0.1410	0.2841	0.6047	0.8906	100.00	0.1291	0.2507	0.5056	0.7951	100.00
Sd	0.0717	0.0320	0.0870	0.0730	5.93	0.0723	0.0311	0.0728	0.0608	7.05
	Weight = 5					Weight = 5				
	BIAS	RMSE	RSE MAX	WIDTH HPD	INSIDE HPD	BIAS	RMSE	RSE MAX	WIDTH HPD	INSIDE HPD
Min	-0.2104	0.0731	0.2066	0.5098	66.00	-0.2104	0.0615	0.1343	0.4736	72.00
Mean	-0.0565	0.1611	0.4364	0.5743	91.98	-0.0461	0.1339	0.3452	0.5205	93.96
Max	0.1110	0.2780	0.7341	1.0585	100.00	0.1111	0.2354	0.4734	0.8205	100.00
Sd	0.0691	0.0375	0.1119	0.0913	6.61	0.0702	0.0333	0.0734	0.0607	5.38
	Weight = 10					Weight = 10				
	BIAS	RMSE	RSE MAX	WIDTH HPD	INSIDE HPD	BIAS	RMSE	RSE MAX	WIDTH HPD	INSIDE HPD
Min	-0.2967	0.0907	0.2690	0.5681	54.00	-0.2572	0.0559	0.1573	0.5050	64.00
Mean	-0.0847	0.1945	0.5134	0.6416	89.56	-0.0742	0.1485	0.3615	0.5511	92.40
Max	0.0970	0.3312	1.0218	1.2108	100.00	0.1193	0.2759	0.5444	0.8494	100.00
Sd	0.0809	0.0453	0.1375	0.1051	8.97	0.0819	0.0431	0.0788	0.0598	7.66
	Weight = 30					Weight = 30				
	BIAS	RMSE	RSE MAX	WIDTH HPD	INSIDE HPD	BIAS	RMSE	RSE MAX	WIDTH HPD	INSIDE HPD
Min	-0.4101	0.1066	0.2877	0.6803	56.00	-0.3770	0.0623	0.1640	0.5362	62.00
Mean	-0.0970	0.2436	0.6307	0.7613	86.74	-0.0684	0.1710	0.3785	0.5757	87.20
Max	0.1275	0.4483	1.0165	1.4042	98.00	0.3421	0.3887	0.7082	0.8335	100.00
Sd	0.1260	0.0639	0.1592	0.1164	9.09	0.1428	0.0787	0.1132	0.0496	7.22

In addition, it is worth noting that the largest difference between true and estimated item difficulties is 0.7082 in the proposed model (shown in Table 7 for weight equal to 30), whereas the largest difference using the standard Rasch model is 1.3339 (shown in Table 5 for weight equal to 30). This improvement is significant since the scale of the item difficulties is based on a standard normal distribution.

Similar to scenario 2, three univariate two-way ANOVA were performed to investigate whether the observed differences between the two models are of statistical significance in scenario 3. The results are shown in Table 8. Assuming a statistical significance level of 5%, it can be concluded that the weight value had a significant effect on the bias, RMSE and RSE_MAX_. Furthermore, the interaction between the estimation method and weight values was statistically significant for RMSE and RSE_MAX_ (p-value is smaller than 5%).

**Table 8:** Two-way ANOVA using data generated in scenario 3.

Two-way ANOVA			
	Estimation method	Weight values	Interaction
**BIAS**	F(1, 490) = 0.369 (p-value = 0.544)	F(4, 490) = 7.180 (**p-value = 0.000**)	F(4, 490) = 0.982 (p-value = 0.417)
**RMSE**	F(1, 490) = 51.96 (**p-value = 0.000**)	F(4, 490) = 39.71 (**p-value = 0.000**)	F(4, 490) = 11.13 (**p-value = 0.000**)
**RSE**_**MAX**_	F(1, 490) = 125.61 (**p-value = 0.000**)	F(4, 490) = 30.14 (**p-value = 0.000**)	F(4, 490) = 22.87 (**p-value = 0.000**)

Bold text indicates significant p-values (< 0.05).

**Fig 5:**
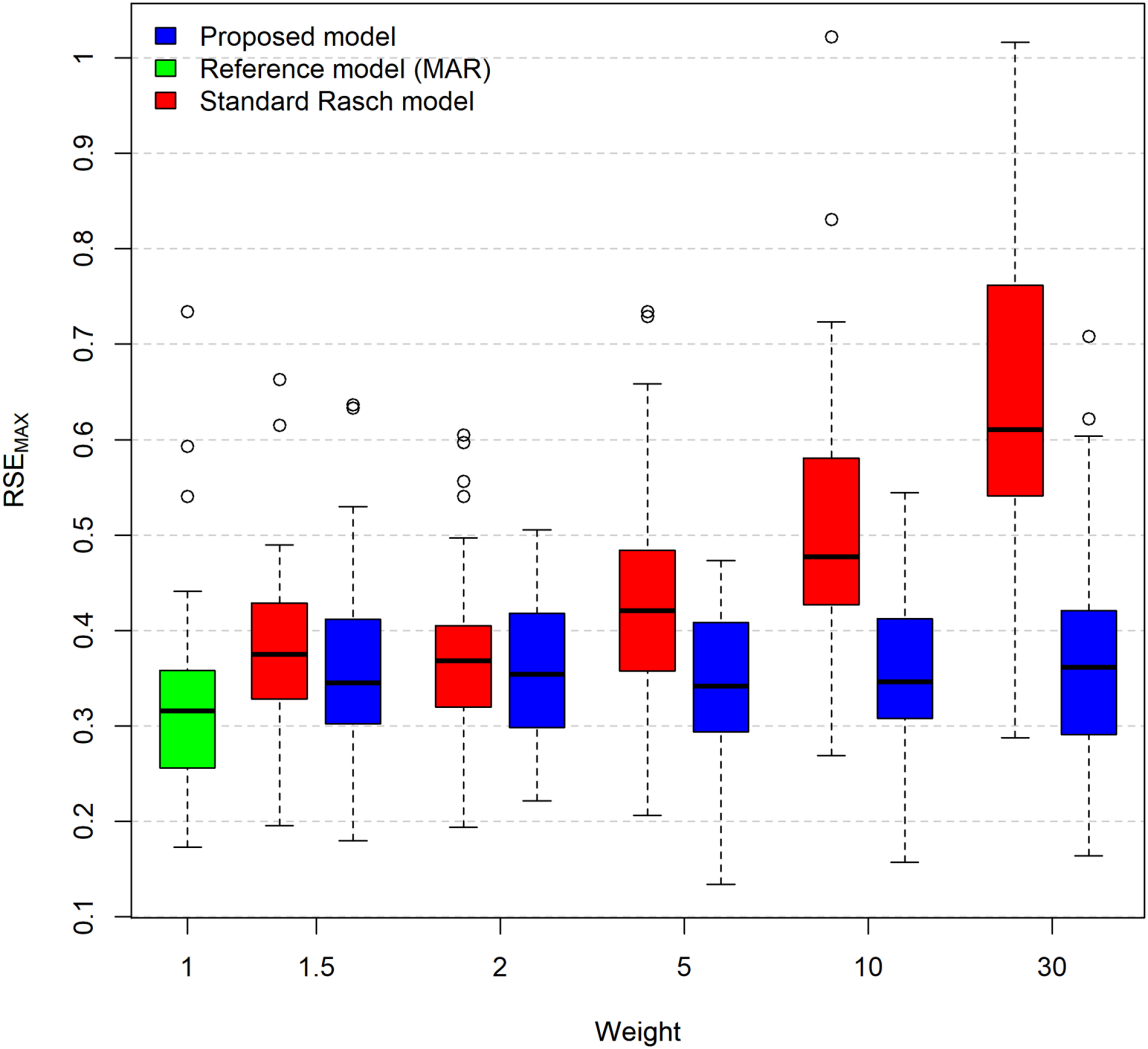
Boxplots of the RSE_MAX_ for all the simulated weights of scenario 3. The blue boxplot represents the results from the proposed model, and the red boxplot represents the results from the standard Rasch model. The green boxplot is used as a reference and was obtained from the adjustment of the standard Rasch model in the data of scenario 1. In the boxplots, the lower outer contour of the rectangle indicates the first quartile (Q1), the upper outer contour of the rectangle indicates the third quartile (Q3), and the horizontal line inside the rectangle indicates the median (Q2). The vertical lines extended from the box indicate variability outside the first and third quartiles. The upper vertical line indicates the maximum observed value within the range [Q3; Q3+1.5×(Q3-Q1)]. The lower vertical line indicates the minimum observed value within the range [Q1; Q1-1.5×(Q3-Q1)]. Observations beyond the vertical lines are represented as points and indicate outliers.

Fig 5 shows the RSE_MAX_ differences among the evaluated models for scenario 3 using different weights. The results of the proposed model are represented by the blue boxplots, and the standard Rasch model results are represented by the red boxplots. Fig 5 also includes the RSE_MAX_ results using the standard Rasch model and scenario 1 (reference), which is represented by the green boxplot. Fig 5 shows that for large values of weights (5, 10 and 30), the proposed model achieves RSE_MAX_ values much closer to the reference scenario than the standard Rasch model results achieve.

Finally, the simulated results using different scenarios, weights and methods were simultaneously compared with the standard Rasch model results (scenario 1) using Dunnett’s test [50]. Dunnett’s test is a multiple t-test comparison procedure that compares the simulation results with the reference scenario (standard Rasch model results for scenario 1). Three statistical tests were evaluated using the bias as the dependent variable, the RMSE as the dependent variable and the RSE_MAX_ as the dependent variable. Table 9 shows the Dunnett’s test results. Using a statistical significance level of 5%, the standard Rasch model results for scenarios 2 and 3 were significantly different from the reference scenario 1 in the following cases: (a) RMSE and RSE_MAX_ in scenario 2 using a weight value of 30, (b) using a weight value of 5 in scenario 3, (c) using a weight value of 10 in scenario 3 and (d) using a weight value of 30 in scenario 3. Using bias as the dependent variable, the results were significantly different in scenario 3 using weight values of 10 and 30. Using RMSE as the dependent variable, our proposed model was significantly different from the reference in scenario 3 using a weight value of 30. It is worth mentioning that for larger values of weights, such as 5, 10, and 30 in scenario 2, larger p-values indicated that our proposed model was closer to the reference than the standard Rasch model regarding RMSE and RSE_MAX_.

**Table 9:** Dunnett’s test p-values comparing each simulated configuration with the reference simulation (Rasch model with scenario 1).

Scenario	Weights	BIAS		RMSE		RSE_MAX_	
		Standard	Proposed	Standard	Proposed	Standard	Proposed
2	1.5	1.000	1.000	0.9762	0.9998	0.831	0.984
	2	1.000	1.000	0.6649	1.000	0.372	1.000
	5	1.000	1.000	0.2124	1.000	0.064	1.000
	10	0.9999	1.000	0.1586	1.000	0.072	1.000
	30	1.000	0.8822	**<0.001**	0.8310	**<0.001**	1.000
3	1.5	1.000	1.000	0.6722	0.7737	0.296	0.707
	2	1.000	0.9124	0.4077	0.4702	0.330	0.828
	5	0.5097	0.9329	**0.0018**	0.9606	**<0.001**	0.999
	10	**0.0089**	0.0571	**<0.001**	0.1040	**<0.001**	0.785
	30	**<0.001**	0.1339	**<0.001**	**<0.001**	**<0.001**	0.280

Bold text indicates significant p-values (< 0.05).

**Fig 6:**
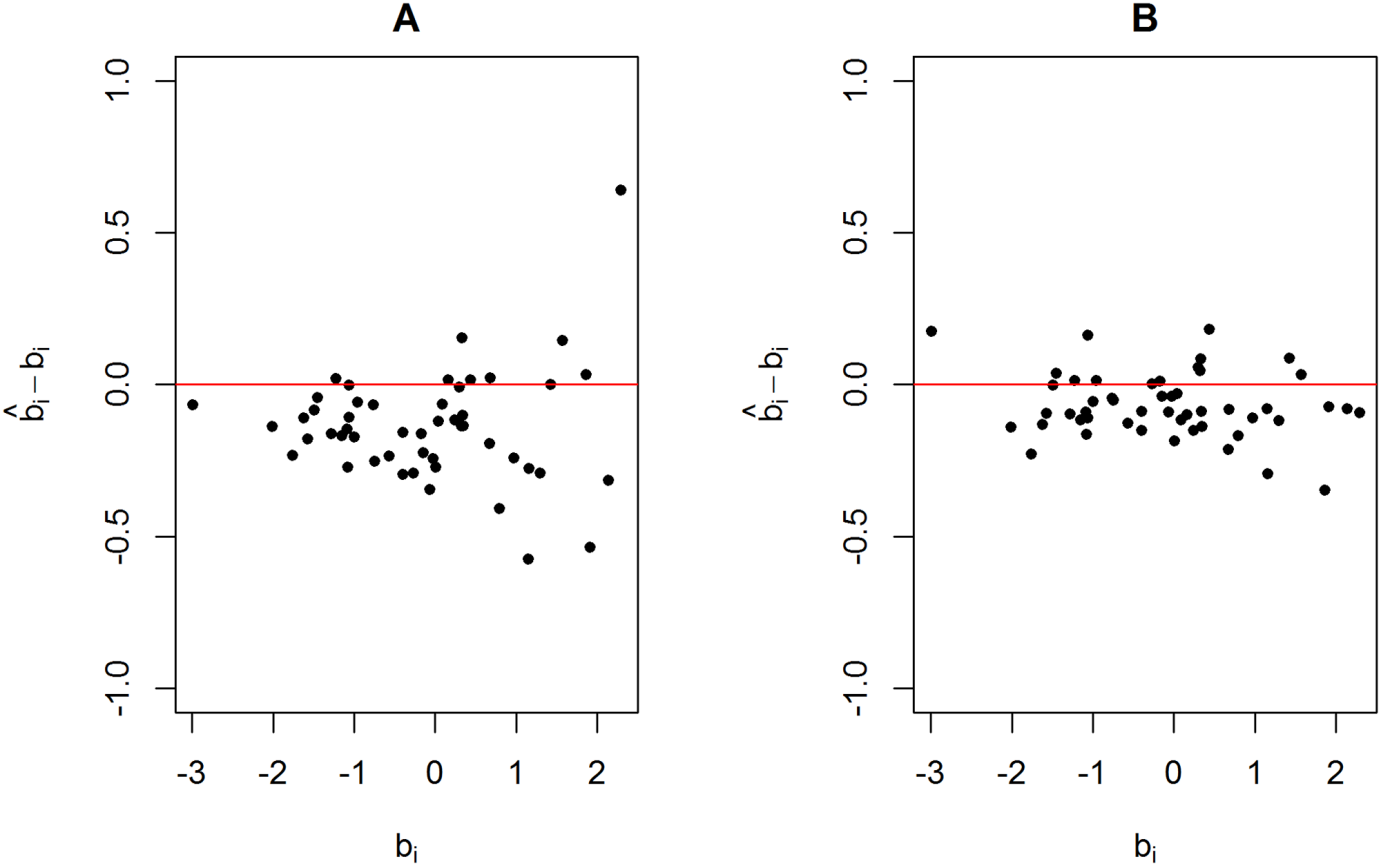
Scatter plot using data generated from scenario 2 with a weight value equal to 10. The differences between estimated and true item difficulties are plotted against the true item difficulties. (A) Estimated values using the standard Rasch model. (B) Estimated values obtained using the proposed model.

Fig 6 shows the differences between the proposed model and the standard Rasch model using a data set generated from scenario 2 with a weight equal to 10. The same data set was used in Fig 3B. Fig 6A presents the residues of the standard Rasch model, and Fig 6B presents the residues of the proposed model. The results shown in Fig 6B seems to be a random cloud centred at zero, as observed in Fig 3A. Furthermore, differences between the true and estimated item difficulties are, in general, lower for the proposed model.

**Fig 7:**
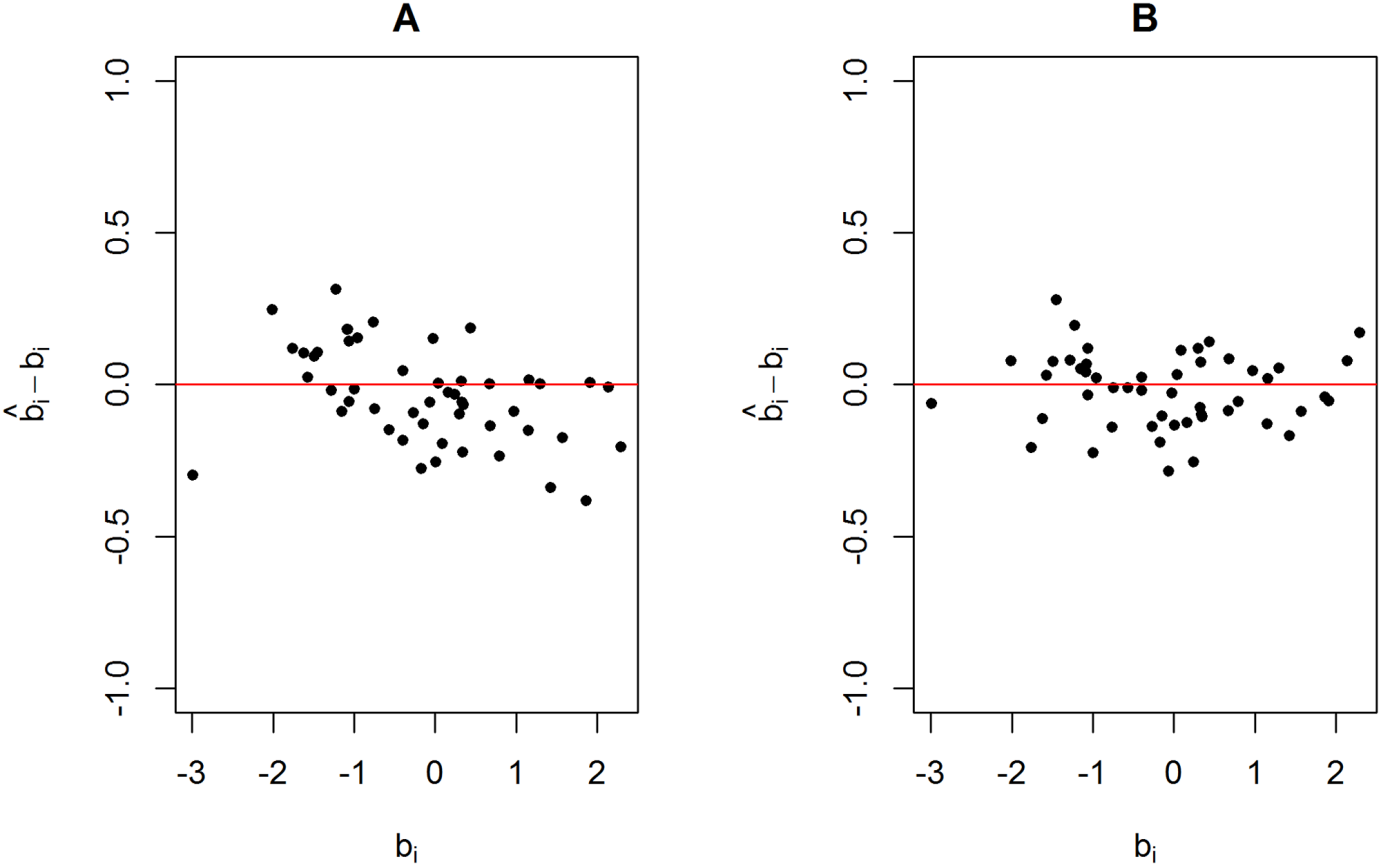
Scatter plot using data generated from scenario 3 with a weight value equal to 10. The differences between the estimated and true item difficulties are plotted against the true item difficulties. (A) Estimated values using the standard Rasch model. (B) Estimated values using the proposed model.

Fig 7 shows the differences between the proposed model and the standard Rasch model using the same data presented in Fig 3C. The proposed two-stage procedure is able to centre the residues at zero. That is, the severe underestimation problem observed in Fig 3C was fixed. Fig 7A shows the results of the standard Rasch model. It is worth noting that these results are the previously estimated results shown in Fig 3C, with the addition of the constant shift. Fig 7B shows the results using the proposed model. The residues of the proposed model are closer to a random cloud centred at zero, as observed in Fig 3A, than the residues of the standard Rasch model.

It is worth mentioning that examinee abilities can be obtained using Bayesian or classical statistical modelling by assuming the item parameter estimates as known values [1] and then estimating the examinee abilities. That is, the estimates of $\vec{\theta }$ can be calculated considering $\vec{b}$ to be a known value in Eq 2. In fact, our proposed model provide better estimates of $\vec{b}$ than the standard model. However, if examinees are free to choose an arbitrary number of items from a questionnaire, the set of selected items may not be appropriate to properly estimate the ability parameter θ_α_. For example, suppose that examinee ability is θ_α_ = 1.5 but the selected items have difficulty parameters (b_i_) below 1. Consequently, the amount of information to estimate θ_α_ is poor [1]. This problem can be overcome by introducing mandatory items. The minimum number of mandatory items is an open research question and will be investigated in future research.

In contrast, if the purpose of the questionnaire is to calibrate items for an item bank rather than estimating the examinee abilities, then the presence of a few mandatory items is required to allow the second stage adjustment in our proposed model. The simulation results showed that a small number of mandatory items, such as two or three mandatory items, is sufficient to provide reliable estimates of the item parameters using the proposed model. As previously described, if some pre-tested items are included in the questionnaire, no mandatory items are required.

## Conclusion

This paper presents a new IRT model to estimate item difficulties using questionnaire data in which examinees are allowed to choose a subset of items. Using network analysis, new information is incorporated in the model. The results are presented using three simulation scenarios. In the first scenario, the assumptions required to apply the standard Rasch model in the literature are met. This scenario is used as the reference scenario. The second and third scenarios include five increasing levels of violating those assumptions. These scenarios are reported in IRT literature, and to the best of our knowledge, no existing proposal provided satisfactory results.

In both scenarios 2 and 3, the standard Rasch model is robust for lower levels of violations. The proposed model performs better in both scenarios, especially for larger levels of violations. Furthermore, the proposed model is able to achieve estimated parameters that are closer to the reference values for both scenarios and different levels of violations, except for the largest violation level in scenario 3. As our main conclusion, we strongly recommend the use of the proposed model over the standard Rasch model when allowing examinees to choose items.

To overcome further technical issues that prevent allowing examinee choice in practical situations, further investigations are required. For instance, real data set analyses are a crucial point for understanding how examinees actually make their choices. Experiments in which examinees are required to indicate which subset of items they would rather answer, despite all items being mandatory, could be used. Furthermore, some important issues such as the number of items and which items should be mandatory (or pre-tested) for the two-stage adjustment were beyond the scope of this paper and require further investigation. Finally, the proposed approach requires further evaluation and research in order to be used in more complex IRT models.

## Supporting information

S1 Table BUGS code for the standard Rasch model. This table shows the BUGS code for the standard Rasch model estimation using MCMC method. Generally, the BUGS code comprises the likelihood function (lines 2–5) and the prior distributions (lines 6–9). It is worth mentioning that the BUGS language uses the precision parameter as opposed to the variance parameter for the normal distribution. Thus, line 10 shows the precision parameter as a function of the standard deviation. Further details about the BUGS code to fit IRT models can be found in [26].
(DOCX)

S2 TableBUGS code for the proposed model.This table shows the BUGS code for the proposed model estimation. Lines 2–5 comprise the likelihood function, lines 6–8 show the hyperparameter prior distributions (β_0_, β_1_ and β_2_), and lines 9–14 show the parameter prior distributions (θ_α_ and b_i_). It is worth noting that line 8 shows that the prior density distribution of β_2_ was truncated on the left at 0.0001 in order to avoid division by zero in Eq 22, that is, β_2_ > 0. Furthermore, in line 12, “rho[i]” represents ρ_i_, and in line 13, "C" represents the lowest value of $\vec{\rho }$; both values are data driven.(DOCX)
