# Supplementary material for: A new item response theory model to adjust data allowing examinee choice
Source: PLoS One. 2018 Feb 1;13(2):e0191600. doi: 10.1371/journal.pone.0191600 (PMC5794135; doi:10.1371/journal.pone.0191600)
Supplement: S1 Table — This table shows the BUGS code for the standard Rasch model estimation using MCMC method. Generally, the BUGS code comprises the likelihood function (lines 2–5) and the prior distributions (lines 6–9). It is worth mentioning that the BUGS language uses the precision parameter as opposed to the variance parameter for the normal distribution. Thus, line 10 shows the precision parameter as a function of the standard deviation. Further details about the BUGS code to fit IRT models can be found in [26]. (DOCX) [file pone.0191600.s001.docx]

S1 Table: BUGS code for the standard Rasch model

| 1 model <- function(){  2 for( alfa in 1 : M ) {  3 for(i in 1:V){  4 y[alfa,i] ~ dbern(prob[alfa,i])  5 logit(prob[alfa,i])<-theta[alfa]-b[i] }}  6 for ( alfa in 1 : M ) {  7 theta[alfa] ~ dnorm(0,1)}  8 for(i in 1:V){  9 b[i] ~ dnorm(m.b,pr.b)}  10 pr.b<-pow(s.b,-2)} |
| --- |
