# Supplementary material for: A new item response theory model to adjust data allowing examinee choice
Source: PLoS One. 2018 Feb 1;13(2):e0191600. doi: 10.1371/journal.pone.0191600 (PMC5794135; doi:10.1371/journal.pone.0191600)
Supplement: S2 Table — This table shows the BUGS code for the proposed model estimation. Lines 2–5 comprise the likelihood function, lines 6–8 show the hyperparameter prior distributions (β0, β1 and β2), and lines 9–14 show the parameter prior distributions (θα and bi). It is worth noting that line 8 shows that the prior density distribution of β2 was truncated on the left at 0.0001 in order to avoid division by zero in Eq 22, that is, β2 > 0. Furthermore, in line 12, “rho[i]” represents ρi, and in line 13, "C" represents the lowest value of ρ→; both values are data driven. (DOCX) [file pone.0191600.s002.docx]

S2 Table: BUGS code for the proposed model

| 1 model <- function(){  2 for( alfa in 1 : M ) {  3 for(i in 1:V){  4 y[alfa,i] ~ dbern(prob[alfa,i])  5 logit(prob[alfa,i])<-theta[alfa]-b[i] }}  6 beta0~dnorm(0,0.001)  7 beta1~dnorm(0, 0.001)  8 beta2~dnorm(0, 0.001)I(0.0001,)  9 for( alfa in 1 : M ) {  10 theta[alfa] ~ dnorm(0, 1)}  11 for(i in 1:V){  12 m.b[i]<- -1/beta1 * (log((1-rho[i] + beta2)/(rho[i]  13 + beta2 - C)) + beta0)  14 b[i] ~ dnorm(m.b[i],pr.b[i]) }  15 pr.b<-pow(s.b,-2)} |
| --- |
